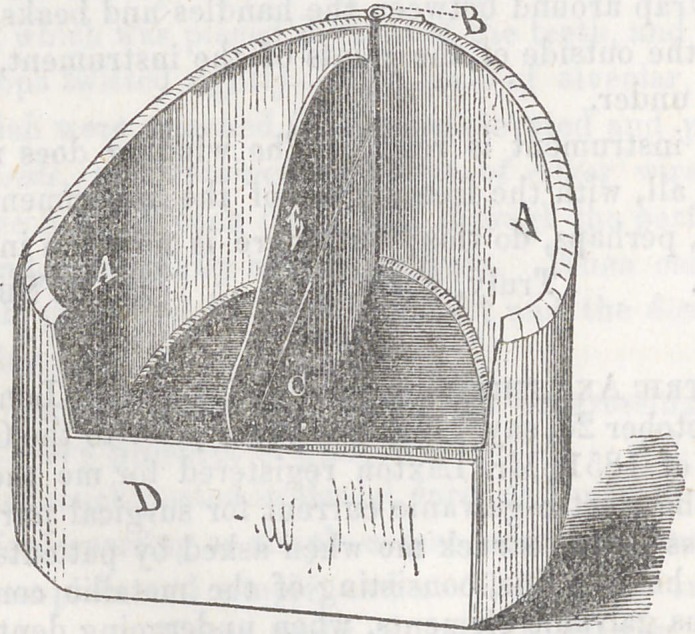# Kellum’s Soldering Flask

**Published:** 1858-12

**Authors:** Wm. C. Kellum

**Affiliations:** Nevada, Cal.


### KELLUM'S SOLDERING FLASK.

The accompanying cuts will enable any one to understand the construction, at a glance. The first one, represents the flask open. The second, closed. *A*, the main of the flask, both edges of which are cut and turned up into a right-angle about one-third of an inch. *B*, is the hinge, by which the two halves of the flask are attached together. *C*, is one of the partitions,—there being two of these, the flask is permitted to operate, by the expansion of the metal, without marring the investment. *D*, is the posterior flange,—there are two of these, and when the flask is closed, these are doubled upon one another.

The flask is a good one, and will undoubtedly be valuable, in the accomplishment of the purpose for which it is designed.

I take the liberty of sending you a soldering flask. I have christened it after myself, which I wish you would place in the Convention, for the benefit of any who may wish to get one made. I sent one to another party, who reported rather unfavorably upon it. Now, all theories must give way to facts; and it is a fact, that a plate will not change, when soldered in this flask. I have used it too long, not to know. Notwithstanding, if a theory is not rational, the fact would not follow. In the April *News Letter* of 1855, Calvert wrote an elaborate report of a series of experiments, made by him, clearly showing, that the plates were changed by the investment, preventing the natural expansion and contraction, under the heat of soldering; his system of expanding the investments with the plate, is, no doubt, a correct one, to prevent its changing; but, to follow it, one must have a furnace, to heat up the entire mass to soldering point,—a matter that persons like me, who travel, do not find convenient. Another thing, there is such an excess of sand,—seven of sand, to one part of plaster,—that you must change your muffles, before you can solder the case, for the reason of its being so tender, you can not back your teeth in it. However, it is an excellent plan, unless there is a better, which I believe this is, as the plate is not confined in any direction in it. J. D. White says, "I presume the expansion to be only across the palatine arch, and that near or at the posterior edge of the plate." Now, I do not presume any such thing. I do presume, however, that that is the only direction the plate is confined in any muffle; it certainly is not along the palatine arch, for there it can always expand backward. Now, aside from theorizing on the subject, all I asked of him, was, to try the thing, and if he could produce any change in the plate, while in the flask, why then I would give it up. But, I judge from his notice of it, that he intends to drop it, because it does not agree with his idea of adaptation to the purpose designed. My experience of the thing, I thought, should have commanded, at least, a trial, before condemning it. If you will put the plate under heat from a blow-pipe, and then turn the case over, you will see how much the plate expands, from the distance the flask will open. It has done me so much good, that I want others to be benefited by it also, as it has saved me many an hour of weary labor. To those who have any better way of doing this, I have nothing to say; but, to those who choose to use one, I pledge my experience of over a year, that they will never have a plate change in soldering,—unless a great amount of solder is used, over the surface. A thin piece of pasteboard is cut, with its edge to fit the palatine portion of the plate, and put between the lips of the flask, in order to divide the investment, and allow it to open under the expansion of the plate.

*Nevada, Cal.* WM. C. KELLUM.

## Figures and Tables

**Figure f1:**
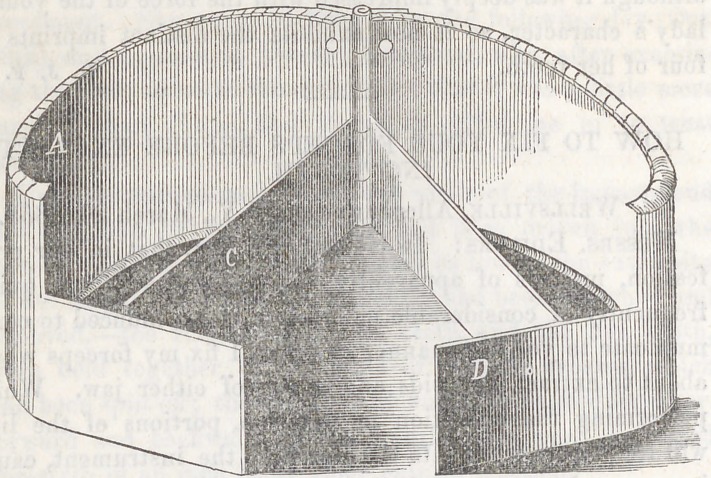


**Figure f2:**